# Content and Color Stability of Anthocyanins Isolated from *Schisandra chinensis* Fruit

**DOI:** 10.3390/ijms131114294

**Published:** 2012-11-05

**Authors:** Chunhui Ma, Lei Yang, Fengjian Yang, Wenjie Wang, Chunjian Zhao, Yuangang Zu

**Affiliations:** State Engineering Laboratory for Bioresource Eco-Utilization, Northeast Forestry University, Harbin 150040, China; E-Mails: mchmchmchmch@163.com (C.M.); yangfj@nefu.edu.cn (F.Y.); wjwang225@hotmail.com (W.W.); zcjsj@163.com (C.Z.)

**Keywords:** *Schisandra chinensis*, anthocyanins, Box-Behnken Design, content stability, color stability, ultrasound treatment, microwave treatment, ultraviolet irradiation

## Abstract

In this work, a multivariate study based on Box-Behnken Design was used to evaluate the influence of three major variables affecting the performance of the extraction process of *Schisandra chinensis* anthocyanins. The optimum parameters were 5.5 h extraction time; 1:19 solid-liquid ratio and 260 r/min stirring rate, respectively. The extraction yield of anthocyanins was 29.06 mg/g under the optimum conditions. Moreover, many factors on the impact of heating, ultrasound, microwave treatment and ultraviolet irradiation on content and color stability of anthocyanins from *Schisandra chinensis* fruit were investigated. The results show that thermal degradation reaction of anthocyanins complies with the first order reaction kinetics, and the correlation coefficient is greater than 0.9950 at 40–80 °C. Ultrasound and microwave treatment has little effect on the stability of anthocyanins, and the extraction time of ultrasound and microwave should be no more than 60 min and 5 min, respectively. The anthocyanins degradation effect of UVC ultraviolet radiation is greater than UVA and UVB; after 9 h ultraviolet radiation, the anthocyanins content degradation of UVC is 23.9 ± 0.7%, and the ΔE* was changed from 62.81 to 76.52 ± 2.3. Through LC-MS analysis, the major composition of *Schisandra chinensis* anthocyanins was cyanidin-3-*O*-xylosylrutinoside.

## 1. Introduction

*Schisandra chinensis* fruit is a well-known traditional medicinal herb and food additive [1]. *S. chinensis* is now widely distributed and cultivated in China, the Far East of Russia, Korea and Japan [2]. Extensive studies have indicated that the major bioactive components of *S. chinensis* fruit are essential oil [3,4] and biphenyl cyclooctene lignans [5–7].

Anthocyanins as a group of flavonoid phenolic compounds widely existing in the plant present a spectrum from orange to blue in color in the natural world, satisfying consumers’ demand for food colors. Anthocyanins have become an important source of natural colorants in cosmetics, food and pharmaceuticals because of the present trend towards replacing synthetic colorants [8]. Chemically, anthocyanins are glycosides of polyhydroxy and polymethoxy derivatives of 2-phenylbenzopyrylium or flavylium salts [9], and correlations with the biosynthesis pathway of flavonol [10]. Because of their polar nature, anthocyanins are soluble in polar solvents, such as acidified methanol, ethanol and water. Recent research has shown that anthocyanins have numerous health beneficial properties, such as prevention of heart disease, inhibition of carcinogenesis [11,12], anti-inflammatory activity [13], antioxidant [14] and free-radical scavenging activity [15]. Hence, there is an increased interest in the use of anthocyanins in functional food, nutraceutical industry food and pharmaceuticals. Anthocyanins have been found in many plant species including strawberries [16], blueberries [17], blackberries [18] black currants [16], black carrot [19], mulberries [20], red grapes [21], purple potatoes [22], red raspberries [23] and so on. Despite their health enhancing properties, no work had been carried out to extract the presence of these colored polyphenols in *S. chinensis* fruit.

In recent years, the stability of food additives and colorant are increasingly concerned. The research is focused on studying the stability of flavonoids [24–29], polyphenolic compounds [30,31] and anthocyanins [24,28,32]. The indicators of stability in the study are content stability [25–29], color stability [32] and the stability of antioxidant capacity [33].

Therefore, in this work, from the point of the reservation of content and color stability to evaluating the degradation of *S. chinensis* anthocyanins, some factors impacting pH, storage temperature and ultraviolet irradiation were investigated. Moreover, the factors in the extraction process, such as ultrasound-assisted and microwave-assisted treatment were also investigated. The results obtained may be used for further optimization studies to design an industrial and commercial production and will be helpful for the full utilization of *S. chinensis* fruit.

## 2. Results and Discussion

### 2.1. Extraction pH

**Figure 1:**
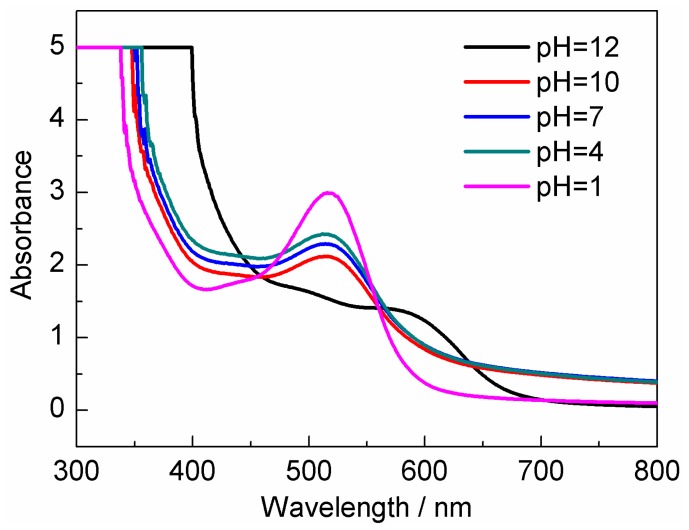
Spectral character of *S. chinensis* fruit anthocyanin at different pH values.

The absorbance and the CIELAB parameters were determined in anthocyanins solutions at different pH values, ranging from 1.0 to 12.0. Modifications in pH were made by addition of small volumes of 0.1 M HCl/NaOH at 25 °C. All samples were spectrophotometrically scanned from 350 to 700 nm. It is observed from Figure 1 that *S. chinensis* fruit anthocyanins extracts had maximum absorption around 510 nm at pH 1 and 4, exhibiting bright-red color. However, when pH values of anthocyanins solutions were above 7, there were no obvious absorption peaks in the 450–600 nm regions. The colors of anthocyanins solutions gradually changed from moderate pink to navy blue from pH 7–12. Moreover, the experimental results also showed that the colors of anthocyanins solutions presented reversible change between acidic and alkaline pH values [34], which was due to the high reactivity of the flavylium cation toward nucleophilic reagents (including OH^−^ and H^+^), resulting in the variation of chemical structures under different pH conditions (this was a reversible pH-dependent reaction) [8]. By the above analysis, in order to maintain their original bright-red color, the best pH value is 1.0 for *S. chinensis* fruit anthocyanins extraction. So, the following experimental condition of pH is 1.0.

### 2.2. Optimization of Extraction Parameters by Box-Behnken Design (BBD)

To study the interaction between the extraction factors on the basis of single-factor experiment, the operating conditions are optimized by Response Surface Methodology (RSM) and use the BBD software in data processing. BBD with three factors is applied using Design-Expert 7.0 without any blocking. The bounds of the factors are 3.0–7.0 h extraction time, 1:10–1:20 g/mL solid-liquid ratio and 180–300 r/min stirring rate. Specific protocols for experimental conditions were shown in Table 1.

To further study the interactions between the factors, we optimized the extraction time, solid-liquid ratio and stirring rate. In Table 2, the Model *F*-value of 45.56 implies the model is significant. There is only a 0.01% chance that a “Model *F*-Value” this large could occur due to noise. Values of “Probability > *F*” less than 0.0001 indicate model terms are very significant, and values of “Probability > *F*” less than 0.0500 indicate model terms are significant. In this case *A*, *B*, *AB*, *A^2^* are significant model terms. Values greater than 0.1000 indicate the model terms are not significant. If there are many insignificant model terms (not counting those required to support hierarchy), model reduction may improve your model.

As can be seen from Table 3, the standard deviation of the model is 0.48. The “Predicted R-Squared” of 0.7321 is in reasonable agreement with the “Adjust R-Squared” of 0.9616. “Adequacy Precision” measures the signal to noise ratio. A ratio greater than four is desirable. This ratio of 23.128 indicates an adequate signal. This model can be used to navigate the design space.

The final extraction yield (*Y*) was given by RSM:

(1) $$Y=28.63+2.55\;A-1.42\;B+0.39\;C+1.27\;AB-0.06\;\text{AC}+0.50\;BC-1.98\;A^{2}-0.53\;B^{2}-0.19\;C^{2}$$

**Figure 2:**
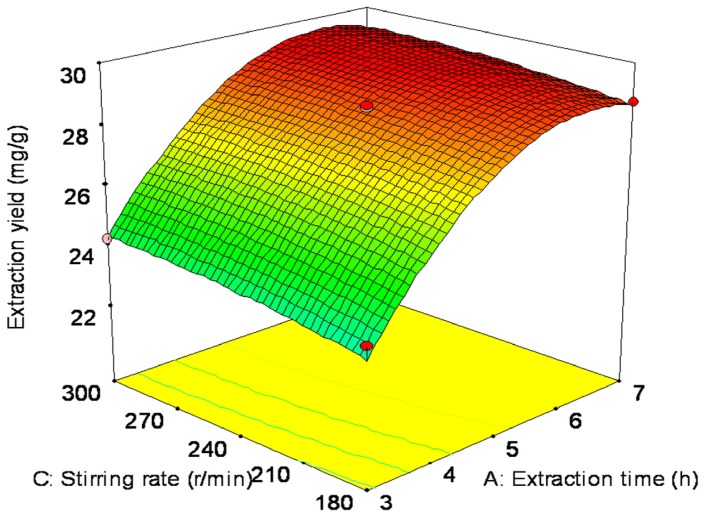
Response surface for the effect of independent variables on extraction yield of *S. chinensis* fruit anthocyanin.

The interaction between factors can clearly be seen through Figure 2. The conditions for point prediction by software were: extraction time 5.57 h with 1:18.87 solid-liquid ratio and 262.04 r/min stirring rate. Under the conditions of point prediction, the extraction yield can reach to 29.66 mg/g.

The verification tests were operated three times under the conditions of point prediction by RSM (extraction time is approximate 5.5 h, solid-liquid ratio and stirring rate are approximate 1:19 and 260 r/min, respectively). The actual extraction yield was 29.06 mg/g, with an error of about 2.0%.

### 2.3. Content and Color Stability of S. chinensis Fruit Anthocyanins

#### 2.3.1. Effect of Heating on Content and Color Stability of Anthocyanins

**Figure 3:**
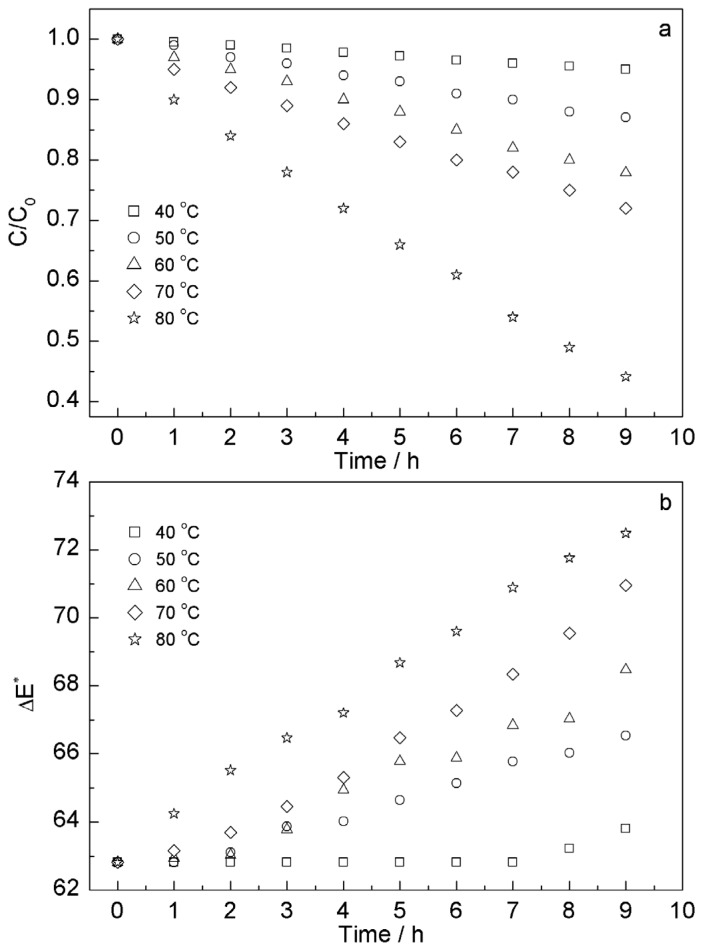
Effect of heating on content (**a**) and color (**b**) stability of *S. chinensis* fruit anthocyanins.

As anthocyanins are not stable at high temperature, heat treatment is one of the most important factors that influence anthocyanin stability. In order to investigate the degradation temperature of anthocyanins, five batches of *S. chinensis* fruit anthocyanins extract were heated in the dark at five different temperatures (40 °C, 50 °C, 60 °C, 70 °C and 80 °C, respectively) by water bath oscillator (HZS-HA, East Union, Harbin, China), each a batch of 10 copies of 50 mL extract and sampled per hour. From Figure 3a, it was observed that the stability of *S. chinensis* fruit anthocyanins in solutions gradually declined with increase of the heating temperature and extension of heating time. Placing the anthocyanins solution for 9 h in water baths at 40 °C, it was stable, and the residual amount was 95.0 ± 0.7%, retained red color and the total color difference (ΔE*) was changed to from 62.81 to 63.81 ± 2.0. However, heated at 50, 60, 70 and 80 °C, the anthocyanins could break down, and their residual amounts rapidly declined within 4 h. After 9 h, the residual amounts were 87.1 ± 0.8% for 50 °C, 77.9 ± 0.6% for 60 °C, 72.0 ± 0.7% for 70 °C and 44.1 ± 0.4% for 80 °C. The ΔE* were 66.54 ± 2.3 for 50 °C, 68.47 ± 1.6 for 60 °C, 70.96 ± 1.7 for 70 °C and 72.49 ± 2.4 for 80 °C from the initial 62.81 in Figure 3b. The results suggested that *S. chinensis* fruit anthocyanins tended to degrade at high temperatures (>50 °C).

Most biological materials and color of food systems follow the zero-order equation or first-order equation reaction [35]:

(2) $$\text{Zero order}:C_{t}=C_{0}+k_{0}\;\text{t}$$

(3) $$\text{First order}:C_{t}=C_{0}\;\text{exp}\;(k_{1}\;\text{t})$$

where *C_t_* and *C_0_* are the anthocyanin content at time t and t_0_, respectively, *k_0_* and *k_1_* are the zero- and first-order kinetic constants, respectively, and t is the storage time (day) [36].

The thermal degradation kinetics of *S. chinensis* fruit anthocyanins is in line with first-order kinetic equation broadly; the first-order kinetic constant (*k*) and correlation coefficient (*r*) are shown in Table 4.

#### 2.3.2. Effect of Ultrasound on Content and Color Stability of Anthocyanins

Ultrasound assisted extraction (UAE) of bioactive compounds was recently reviewed by Vilkhu *et al.*[37]. Applications of ultrasound in food processing are reviewed by Knorr *et al.*[38]. Literature indicates ultrasound processing improves the extraction yield of bioactive compounds [37]. Ultrasound processing is reported to have a minimal effect on the degradation of key quality parameters such as color and anthocyanins content of juices [39,40]. So, the influence of ultrasound processing on the stability of *S. chinensis* fruit anthocyanins was investigated in the dark with an ultrasonic bath (KQ-250DB, Kunshan Ultrasonic Co. Ltd., Jiangsu, China) to which 50 kHz transducers were annealed at the bottom. The bath power rating is 250 W, and the water temperature in the bath is controlled at 25 °C. The characteristics of anthocyanins solution used for ultrasound are listed in Table 5. The degradation time of ultrasound is in the range of 0–90 min; nine copies of 50 mL extract was sampled every 10 min. When the treat time of ultrasound is 60 min, the residual amount of *S. chinensis* fruit anthocyanin is 95.0 ± 0.6%, and the ΔE* is changed from initial 62.81 to 66.95 ± 1.7. While the treat time of ultrasound is 90 min, the residual amount of anthocyanins is 93.0 ± 0.4% and the ΔE* is changed from initial 62.81 to 67.88 ± 2.7. The degradation of anthocyanins during the ultrasound process may be due to the extreme physical conditions that occur within the bubbles during cavitational collapse at micro-scale and several sonochemical reactions occurring simultaneously or in isolation [41]. When extracting of anthocyanins with the assistance of ultrasound, the preferred ultrasound treatment time is not more than 60 min.

#### 2.3.3. Effect of Microwave on Content and Color Stability of Anthocyanins

In recent years, microwave-assisted extraction (MAE) technology developed rapidly, especially in extraction of natural products [42]. Many applications of microwave in extraction of anthocyanins are reported [43,44]. However, the stability of anthocyanins in the process of microwave-assisted extraction has not been mentioned. In this study, the influence of microwave processing on the stability of *S. chinensis* fruit anthocyanins was investigated by a microwave oven (PJ21C-AU, Guangdong, China). Its maximum output power is 700 W. The characteristics of anthocyanins solution used for microwave are listed in Table 5. The degradation time of microwave is in the range of 0–45 min; nine copies of 50 mL extract were sampled every 5 min. When the microwave treatment time is 5 min, the residual amount of *S. chinensis* fruit anthocyanin is 95.0 ± 0.6%, and the ΔE* is changed from initial 62.81 to 67.79 ± 2.6. When the microwave treatment time is 45 min, the residual amount of anthocyanins is 90.0% ± 0.8%, the ΔE* is changed from initial 62.81 to 70.65 ± 2.5. In the process of conventional heating, the heat transfer is mainly performed by conduction and convection only; in contrast, in the MAE process, the heat transfer is performed in three ways—radiation, conduction and convection [45]. As a result, heat is produced from within cells as well as from the outside. So, the thermal degradation of *S. chinensis* fruit anthocyanins also exists because of the fast heating in the microwave-assisted process. When extracting anthocyanins with the microwave-assisted process, the microwave treatment time is preferably not more than 5 min.

#### 2.3.4. Effect of Ultraviolet Irradiation on Content and Color Stability of Anthocyanins

**Figure 4:**
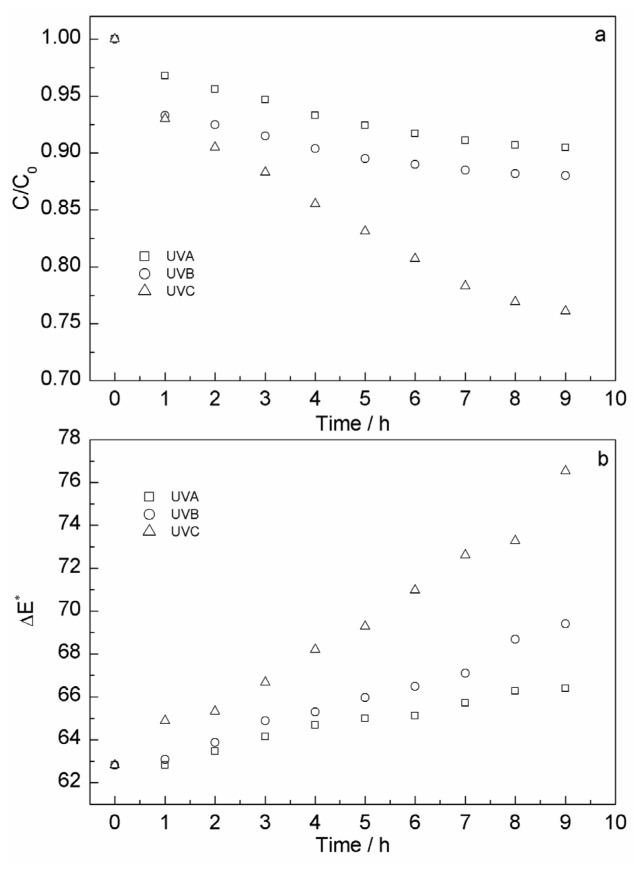
Effect of ultraviolet irradiation on content (**a**) and color (**b**) stability of *S. chinensis* fruit anthocyanins.

Recently, there has been a considerable interest in non-thermal technologies for preservation of fresh-like, high quality and nutritious food products [46]. UV irradiation is a very important non-thermal factor for content and color stability of anthocyanins. The effect of UVC light on anthocyanins content of pomegranate juice was studied by Pala *et al.*[47]. The effect of UV radiation on kinetic degradation of anthocyanins mixtures extracted from *Hibiscus acetosella* was studied by Mar *et al.*[48]. And, the effect of gamma irradiation on decolorization and biological activity in *S. chinensis* fruit extracts was studied by Lee *et al.*[49]. However, there is no study on the effect of UVA, UVB and UVC treatment on the *S. chinensis* fruit anthocyanins solution. In this study, the influence of UV irradiation on the stability of anthocyanins was investigated. The ultraviolet strip lamp (S9W/01, Philips, Holland), including UVA (320–400 nm), UVB (280–320 nm) and UVC (200–280 nm) was used for UV irradiation 9 h, and nine copies of 50 mL extract was sampled per hour. The intensity was 4.5 ± 0.5 μmol·m^−2^·s^−1^ during the irradiation degradation process. From Figure 4, the content of anthocyanins decreases with increasing irradiation time for all ultraviolet strip lamps. There was a strong degradation effect of UVC on content and color stability of *S. chinensis* fruit anthocyanins. After 9 h, the residual amount is 76.1% ± 0.7%, and the ΔE* is 76.52 ± 2.3. However, UVC is rare in the sun’s ultraviolet spectrum; compared with UVC, UVA and UVB had more influence on the stability of antioxidants in our daily life. In contrast, with UVA and UVB degradation for 9 h, the residual amounts are 90.4% ± 0.5%, 87.3% ± 0.6%, and the ΔE* are 66.22 ± 2.5, 69.15 ± 2.3, respectively.

#### 2.3.5. LC-MS Analysis of *S. chinensis* Major Anthocyanins

**Figure 5:**
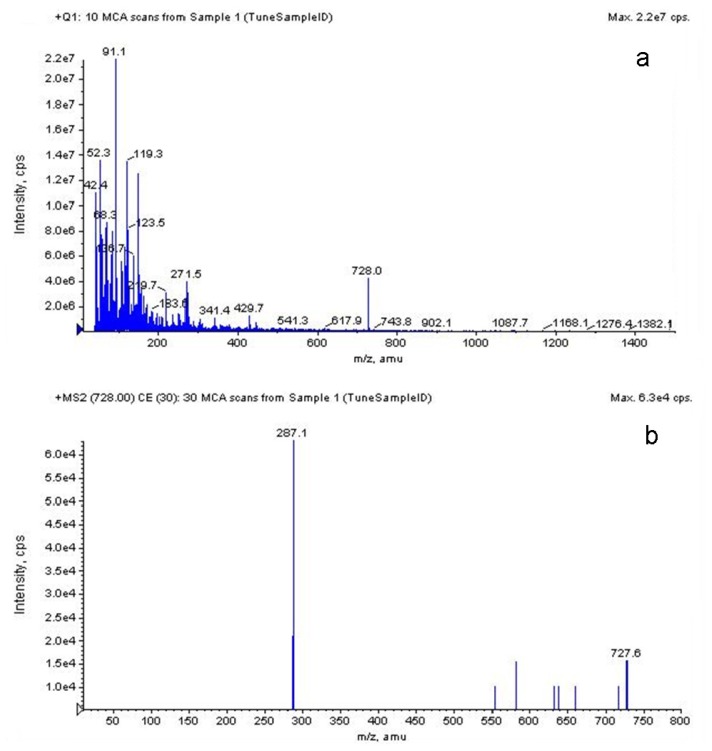

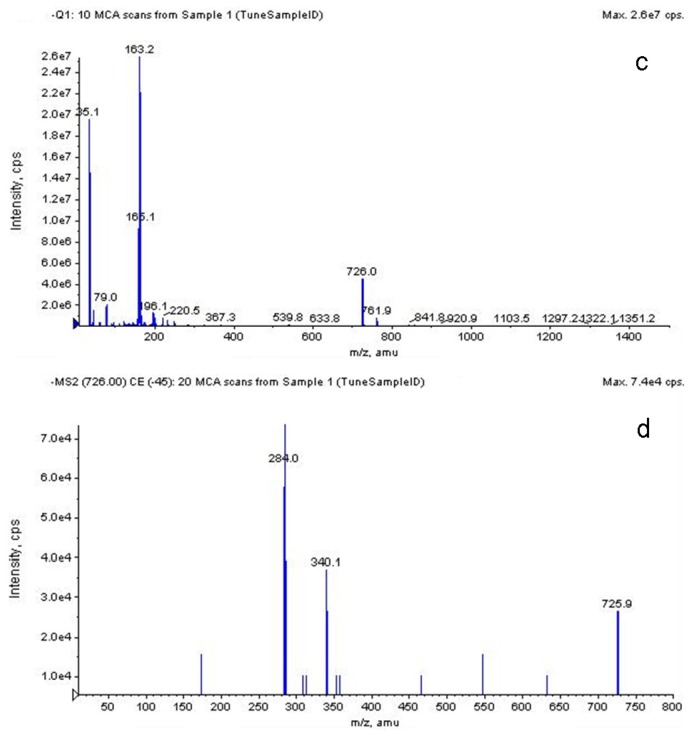
HPLC-MS Chromatograms of Cyanidin-3-*O*-xylosylrutinoside. (**a**) MS1 chromatograms of cyanidin-3-*O*-xylosylrutinoside under ESI-MS in positive mode; (**b**) MS2 chromatograms of cyanidin-3-*O*-xylosylrutinoside under ESI-MS in positive mode; (**c**) MS1 chromatograms of cyanidin-3-*O*-xylosylrutinoside under ESI-MS in negative mode; (**d**) MS2 chromatograms of cyanidin-3-*O*-xylosylrutinoside under ESI-MS in negative mode.

A major HPLC peak of *S. chinensis* anthocyanin represented about 95% of total absorbable compounds at 520 nm, and the mass-to-charge ratio (*m*/*z*) and molecular weight of the major anthocyanin was determined to be 727 [50]. As a result, it was confirmed that the relative intensity of the cyanidin-3-*O*-xylosylrutinoside (Cya-3-*O*-xylrut; *m*/*z* 727) decreased with an increase in fragmentor voltage, whereas the m/z 287 assigned to cyanidin was generated. Moreover, another fragmented ion molecule was detected, and its molecular weight was exactly correspondent with [M-rhamnose]+. The β-(1,6) linkage between glucose and rhamnose residues may be weaker than other glycosidic bonds in Cya-3-*O*-xylrut, which thus resulted in Cya-3-*O*-rut during the fragmentation. HPLC-MS Chromatograms of Cyanidin-3-*O*-xylosylrutinoside were show in Figure 5. According to a few studies, Cya-3-*O*-xylrut was one of the major anthocyanins in red current [51,52]. Thus, *S. chinensis* may be a unique source of highly pure Cya-3-*O*-xylrut.

## 3. Experimental Section

### 3.1. Material

*S. chinensis* fruit was purchased from San Keshu Trading (Heilongjiang, China) and identified by Prof. Shao-quan Nie from Key Laboratory of Forest Plant Ecology, Northeast Forestry University. All the reagents obtained from Beijing Chemical Reagents Co. (Beijing, China) were of analytical grade. Deionized water was purified by a Milli-Q water purification system from Millipore (Bedford, MA, USA).

### 3.2. Methods

#### 3.2.1. The Extract Samples Preparation

The same batch of sample was dried naturally, and used here in the experiments. The samples were sieved by 40–60 mesh after crushing, and then stir-extracted with 1.0 M hydrochloric acid under the optimized conditions obtained by RSM. The supernatant fluid after filtering through a 0.45 μm membrane was extracted 2 times with the same volume of petroleum ether, removing the fat-soluble impurities, and extracted 2 times with the same volume of ethyl acetate. Then the water layer was used for future stability experiments.

#### 3.2.2. Total Monomeric Anthocyanins (TMA)

Total anthocyanin content was measured using the pH differential method with minor modifications by UV-2550 UV-Vis spectrophotometer (Shimadzu, Japan). The crude anthocyanin extracts were dissolved in potassium chloride buffer (KCl, 0.025 M, pH 1.0) and sodium acetate (CH_3_COONa·3H_2_O, 0.4 M, pH 4.5) with a pre-determined dilution factor. The absorbances of measured samples were read at 510 nm and 700 nm against a blank cell containing deionized water. The absorbance (*A*) of the diluted sample was then calculated as follows:

(4) $$A={\left(A_{510\;\text{nm}}-A_{700\;\text{nm}}\right)}_{\text{pH }1.0}-{\left(A_{510\;\text{nm}}-A_{700\;\text{nm}}\right)}_{\text{pH }4.5}$$

The monomeric anthocyanin pigment concentration in the original sample was calculated according to the following formula:

(5) Anthocyanin content (mg/mL)=A·MW·DF·1000/(ɛ·1)

where the molecular weight of main anthocyanin (cyanidin-3-glucoside *MW* = 449.2), the dilution factor or dilution multiple (*DF* = 5) and the molar absorbtivity constant of main anthocyanin (cyanidin-3-glucoside ɛ = 26,900) were used.

#### 3.2.3. Colorimetric Study

The colorimeter study of samples was evaluated by TCP2 automatic colorimeter (Aoyike, Beijing, China). (L* *a* *b *) (CIELAB) uniform color space was taken into account for the colorimetric analysis. Within the CIELAB uniform space, a psychometric index of lightness, *L* * (ranging from 0, black, to 100, white) and two color coordinates—a* (which takes positive values for reddish colors and negative values for greenish ones) and b* (positive for yellowish colors and negative for the bluish ones)—are defined. From these coordinates, other color parameters are defined: the hue angle (h*ab) is the qualitative attribute of color and the chroma (*C* *ab) is the quantitative attribute of color intensity [53]. Total color difference (ΔE*) expressed the magnitude of difference between the initial non-aged juice (zero time) and storage-aged samples. Total color difference (ΔE*) was calculated according to the following formula [54]:

(6) $$\Delta E^{*}={\left(\Delta {L^{*}}^{2}+\Delta {a^{*}}^{2}+\Delta {b^{*}}^{2}\right)}^{1/2}$$

where Δa* = a* − *a*_0_*, Δb* = b* − *b*_0_*, ΔL* = L* − *L*_0_*; *a*_0_*, *b*_0_* and *L*_0_* are the corresponding blank values of control sample, and *a*_0_* = −2.00, *b*_0_* = 4.00 and *L*_0_* = 80.00, respectively. All trials were carried out in triplicate, and data were reported as mean ± standard deviation (SD, *n* = 3). The statistical significance was evaluated using Microsoft Excel 2003 and set at *p* < 0.05.

#### 3.2.4. LC-UV-ESI-MS Analysis

The water layer after extraction obtained from Section 3.2.1 was purified by macroporous resin HPD-300 column chromatography, removing the carbohydrates, proteins, organic acids and other water-soluble impurities with distilled water elution. HPD-300 was eluted by 0.1% HCl methanol solution, concentrated under vacuum for dryness, and then dissolved in 10% methanol and used for structure determination of *S. chinensis* fruit anthocyanins by LC-UV-ESI-MS.

An Agilent 1100 series HPLC system equipped with G1312A Bin pump, a G1379A Degasser (Agilent, San Jose, CA, USA), a G1316A automatic column temperature control box and a 2487 UV-detector (Waters, USA). Chromatographic separation was performed on a HiQ sil-C18 reversed-phase column (4.6 mm × 250 mm, 5 μm, KYA TECH). Three-percent acetic acid solution was used as eluent A, and HPLC-grade acetonitrile was used as eluent B. The gradient profile began at 10%–15% B at 40 min, 20% B at 45 min, and subsequently, then isocratic with 20% B for 20 min, and then returned to initial conditions at 65 min and for 5 min. The flow rate was 1.0 mL/min, and the column temperature was maintained at 25 °C. The injection volume was 10 μL, and the detection wavelength was 520 nm.

An API3000 Triple tandem quadrupole mass spectrometry with a Turbolon-Spray interface from Applied Biosystems (CA, USA) was operated in positive and negative electro spray ionization (ESI, *m*/*z* 100–1000) source mode. All mass spectra were acquired in multiple reaction monitoring transitions. The analytical conditions were as follows: the ion source was operated at a temperature of 250 °C; The nebulising gas and nebulizer pressure was 380 Pa; The ion spray voltage was 4500 V; The entrance potential and focusing potential were set at 10 and 375 V; The declustering’s potential was 80. Analyst software (version 1.4) installed on a Dell computer was used for data acquisition and processing.

## 4. Conclusions

In the present study, changes in color of anthocyanins have been evaluated over pH range 1.0–12.0. Under acidic conditions (pH = 1.0), the red color stability of anthocyanins is better. Under the optimum conditions based on Box-Behnken Design, 5.5 h extraction time, 1:19 solid-liquid ratio and 260 rpm stirring rate, respectively, and the extraction yield of anthocyanins was 29.06 mg/g. The results of content and color stability of *S. chinensis* fruit anthocyanins show that thermal degradation reaction of anthocyanins complies with the first order reaction kinetics, and the correlation coefficient is greater than 0.9950 at 40–80 °C. In the extraction process of anthocyanins, the degradation effect of microwave-assisted extraction is greater than that of ultrasound-assisted. Moreover, there is great impact of the different wavelengths of ultraviolet irradiation on the stability of anthocyanins. The anthocyanins degradation effect of UVC ultraviolet radiation is greater than UVA and UVB; after 9 h ultraviolet radiation, the anthocyanins content degradation of UVC is 23.9% ± 0.7%, and the ΔE* was changed from 62.81 to 76.52 ± 2.3. The result of LC-MS showed that the major composition of *S. chinensis* anthocyanins was cyanidin-3-*O*-xylosylrutinoside.

## Figures and Tables

**Table 1:** Experimental design matrix to screen important variables for extraction yield of anthocyanins.

Run	Factor A	Factor B	Factor C	Response

	Extraction time (h)	Solid-liquid ratio (g/mL)	Stirring rate (r/min)	Extraction yield (mg/g)
1	3.0	1:20	240	26.43
2	3.0	1:15	180	23.86
3	3.0	1:10	240	20.37
4	5.0	1:20	300	29.13
5	5.0	1:15	240	28.61
6	7.0	1:10	240	28.33
7	7.0	1:15	300	28.92
8	7.0	1:20	240	29.30
9	5.0	1:15	240	28.60
10	5.0	1:10	300	27.97
11	5.0	1:20	180	28.84
12	5.0	1:10	180	25.66
13	5.0	1:15	240	28.60
14	5.0	1:15	240	28.67
15	5.0	1:15	240	28.65
16	3.0	1:15	300	24.25
17	7.0	1:15	180	28.77

**Table 2:** Test of significance for regression coefficient a.

Source	Sum of squares	Df	Mean square	*F*-value	*p*-Value
Model b	95.74	9	10.64	45.56	<0.0001
A	52.07	1	52.07	223.02	<0.0001
B	16.16	1	16.16	69.21	<0.0001
C	1.23	1	1.23	5.28	0.0552
AB	6.48	1	6.48	27.74	0.0012
AC	0.014	1	0.014	0.062	0.8110
BC	1.02	1	1.02	4.37	0.0749
A^2^	16.58	1	16.58	71.00	<0.0001
B^2^	1.20	1	1.02	5.15	0.0576
C^2^	0.15	1	0.15	0.66	0.4423
Linear	27.91	9	3.10	3010.56	<0.0001
2FI	20.40	6	3.40	3300.38	<0.0001
Quadratic	1.63	3	0.54	527.60	<0.0001
Cubic	0.00	0	-	-	-
Residual	1.63	7	0.23	-	-
Lack of fit	1.63	3	0.54	527.60	<0.0001
Pure Error	4.12 × 10^−3^	4	1.03 × 10^−3^	-	-
Cor Total	97.38	16	-	-	-

a The results were obtained with the Design Expert 7.0 software;

b A is extraction time (h), B is solid-liquid ratio (g/mL) and C is stirring rate (r/min).

**Table 3:** Credibility analysis of the regression equations.

Index mark^a^	Values
Std. Dev.	0.48
Mean	27.35
C.V. %	1.77
PRESS	26.09
R-Squared	0.9832
Adjust R-Squared	0.9616
Predicted R-Squared	0.7321
Adequacy Precision	23.128

a The results were obtained with the Design Expert 7.0 software.

**Table 4:** Thermal degradation kinetic parameters of *S. chinensis* anthocyanin.

Temperature (°C)	First-order kinetic equation	Kinetic constant (*k*)	Correlation coefficient (*r*)
40	*Y* = − 0.0057 *X* + 1.0066	0.0057	0.9988
50	*Y* = − 0.0148 *X* + 1.0165	0.0148	0.9982
60	*Y* = − 0.0248 *X* + 1.0242	0.0248	0.9989
70	*Y* = − 0.0298 *X* + 1.0140	0.0298	0.9969
80	*Y* = − 0.0604 *X* + 1.0305	0.0604	0.9974

**Table 5:** Effect of extraction time on content and color stability of *S. chinensis* anthocyanins.

Treat time of ultrasonication (min)	C/C_0_^a^ ± SD b (%)	ΔE^*^ c ± SD b
0	1.000	62.81
10	98.0 ± 0.7	65.17 ± 2.5
20	97.0 ± 0.7	65.82 ± 2.6
30	96.0 ± 0.5	66.35 ± 1.7
40	96.0 ± 0.4	66.45 ± 2.5
50	96.0 ± 0.7	66.54 ± 2.4
60	95.0 ± 0.6	66.95 ± 1.7
70	94.0 ± 0.6	67.38 ± 2.4
80	94.0 ± 0.6	67.86 ± 2.6
90	93.0 ± 0.4	67.88 ± 2.7

**Treat time of microwave (min)**	**C/C_0_**^a^**± SD**^b^**(%)**	ΔE* c**± SD**^b^

0	1.000	62.81
5	95.0 ± 0.6	67.79 ± 2.6
10	94.0 ± 0.7	67.92 ± 2.4
15	93.0 ± 0.8	68.57 ± 2.7
20	93.0 ± 0.4	68.90 ± 2.7
25	92.0 ± 0.4	69.07 ± 2.4
30	91.0 ± 0.5	69.26 ± 2.4
35	91.0 ± 0.6	69.86 ± 1.7
40	90.0 ± 0.7	70.49 ± 1.5
45	90.0 ± 0.8	70.65 ± 2.5

a C/C_0_: the ratio of degradation concentration and initial concentration;

b SD: standard deviation;

c ΔE*: total color difference.
